# The multicenter effectiveness study of inpatient and day hospital treatment in departments of psychosomatic medicine and psychotherapy in Germany

**DOI:** 10.3389/fpsyt.2023.1155582

**Published:** 2023-08-07

**Authors:** Stephan Doering, Stephan Herpertz, Magdalena Pape, Tobias Hofmann, Matthias Rose, Katrin Imbierowicz, Franziska Geiser, Antonie Louise Bierling, Kerstin Weidner, Jörg Rademacher, Silke Michalek, Eva Morawa, Yesim Erim, Per Teigelack, Martin Teufel, Armin Hartmann, Claas Lahmann, Eva Milena Johanne Peters, Johannes Kruse, Dirk von Boetticher, Christoph Herrmann-Lingen, Mariel Nöhre, Martina de Zwaan, Ulrike Dinger, Hans-Christoph Friederich, Alexander Niecke, Christian Albus, Rüdiger Zwerenz, Manfred Beutel, Heribert Christian Sattel, Peter Henningsen, Barbara Stein, Christiane Waller, Karsten Hake, Carsten Spitzer, Andreas Stengel, Stephan Zipfel, Katja Weimer, Harald Gündel, Henrik Kessler

**Affiliations:** ^1^Department of Psychoanalysis and Psychotherapy, Medical University of Vienna, Vienna, Austria; ^2^Comprehensive Clinical Center for Neurosciences and Mental Health (C_3_NMH), Medical University of Vienna, Vienna, Austria; ^3^Department of Psychosomatic Medicine and Psychotherapy, LWL-University Hospital, Ruhr-University Bochum, Bochum, Germany; ^4^Charité Center for Internal Medicine and Dermatology, Department of Psychosomatic Medicine, Charité-Universitätsmedizin Berlin, Corporate Member of Freie Universität Berlin and Humboldt-Universität zu Berlin, Berlin, Germany; ^5^Department of Psychosomatic Medicine and Psychotherapy, University of Bonn, Bonn, Germany; ^6^Department of Psychotherapy and Psychosomatic Medicine, Carl Gustav Carus Faculty of Medicine, Technische Universität, Dresden, Germany; ^7^Institute for Material Science and Nanotechnology, Technical University of Dresden, Dresden, Germany; ^8^Department of Clinical Psychology, Friedrich-Schiller University, Jena, Germany; ^9^Department of Psychosomatic Medicine and Psychotherapy, LVR-University Hospital, Heinrich Heine University Düsseldorf, Düsseldorf, Germany; ^10^Department of Psychosomatic Medicine and Psychotherapy, University Hospital of Erlangen, Friedrich-Alexander University Erlangen-Nuremberg, Erlangen, Germany; ^11^Clinic of Psychosomatic Medicine and Psychotherapy, LVR-University Hospital, University of Duisburg-Essen, Essen, Germany; ^12^Department of Psychosomatic Medicine und Psychotherapy, Center for Mental Health, Faculty of Medicine, University of Freiburg, Freiburg, Germany; ^13^Department of Psychosomatic Medicine and Psychotherapy, Justus-Liebig University of Giessen, Giessen, Germany; ^14^Department of Psychosomatic Medicine and Psychotherapy, Philipps-University of Marburg, Marburg, Germany; ^15^Department of Psychosomatic Medicine and Psychotherapy, University of Göttingen Medical Centre, Göttingen, Germany; ^16^Department of Psychosomatic Medicine and Psychotherapy, Hannover Medical School, Hannover, Germany; ^17^Department of General Internal Medicine and Psychosomatics, University Hospital, Heidelberg University, Heidelberg, Germany; ^18^Department of Psychosomatic Medicine and Psychotherapy, University of Cologne, Faculty of Medicine and University Hospital Cologne, Cologne, Germany; ^19^Department of Psychosomatic Medicine and Psychotherapy, University Medical Center of the Johannes Gutenberg University Mainz, Mainz, Germany; ^20^Department of Psychosomatic Medicine and Psychotherapy, University Hospital, Technical University of Munich, Munich, Germany; ^21^Department of Psychosomatic Medicine and Psychotherapy, Paracelsus Medical University, Nuremberg General Hospital, Nuremberg, Germany; ^22^Department of Psychosomatic Medicine and Psychotherapy, University Medical Center Rostock, Rostock, Germany; ^23^Internal Medicine VI, Psychosomatic Medicine and Psychotherapy, University Hospital Tübingen, Tübingen, Germany; ^24^Department of Psychosomatic Medicine and Psychotherapy, Ulm University Medical Center, Ulm, Germany; ^25^Department of Psychosomatic Medicine and Psychotherapy, Campus Fulda, University of Marburg, Marburg, Germany

**Keywords:** inpatient, psychosomatic treatment, eating disorders, somatoform disorders, personality functioning

## Abstract

**Background:**

Reliable outcome data of psychosomatic inpatient and day hospital treatment with a focus on psychotherapy are important to strengthen ecological validity by assessing the reality of mental health care in the field. This study aims to evaluate the effectiveness of inpatient and day hospital treatment in German university departments of Psychosomatic Medicine and Psychotherapy in a prospective, naturalistic, multicenter design including structured assessments.

**Methods:**

Structured interviews were used to diagnose mental disorders according to ICD-10 and DSM-IV at baseline. Depression, anxiety, somatization, eating disorder and posttraumatic stress disorder (PTSD) symptoms, as well as personality functioning were assessed by means of questionnaires on admission and at discharge.

**Results:**

2,094 patients recruited by 19 participating university hospitals consented to participation in the study. Effect sizes for each of the outcome criteria were calculated for 4–5 sub-groups per outcome domain with differing severity at baseline. Pre-post effect sizes for patients with moderate and high symptom severity at baseline ranged from *d* = 0.78 to *d* = 3.61 with symptoms of PTSD, depression, and anxiety showing the largest and somatization as well as personality functioning showing somewhat smaller effects.

**Conclusions:**

Inpatient and day hospital treatment in German university departments of Psychosomatic Medicine and Psychotherapy is effective under field conditions.

**Clinical trial registration:**

https://drks.de/search/de/trial/DRKS00016412, identifier: DRKS00016412.

## 1. Introduction

Evidence for the effectiveness of inpatient and day hospital psychosomatic treatment with a focus on psychotherapy is limited, particularly when it comes to longer-term treatment. This can be attributed to the fact that in many countries longer-term psychosomatic hospital treatment is not covered by health insurances. Moreover, methodological, pragmatic, and ethical reasons complicate randomization and control groups.

A high number of psychosomatic inpatient units is provided by the German health care system. These are predominantly located in hospitals and departments of Psychosomatic Medicine and Psychotherapy, which in Germany represents a medical specialty in its own. In 2020, 278 departments and hospitals provided 12,773 beds for inpatient treatment in Psychosomatic Medicine and Psychotherapy (1) (Germany has a population of 84 million).

Inpatient psychosomatic treatment in Germany is guideline-oriented in accordance with the criteria of the Association of Scientific Medical Societies in Germany (AWMF) with a treatment focusing on high intensity psychotherapeutic approaches. The specialty of Psychosomatic Medicine and Psychotherapy comprises almost the complete spectrum of mental disorders, particularly, somatoform/functional disorders, eating disorders, trauma-related disorders, psychosomatic aspects in somatic diseases (e.g., heart diseases and cancer, diabetes), personality disorders, and affective disorders; patients with acute psychoses, severe organic brain disorders, or severe substance-related disorders are usually not admitted (2). Different from outpatient treatment, inpatient as well as day hospital psychosomatic treatment takes place in a multimodal treatment setting, i.e., the combination of a variety of therapeutic approaches for a duration of 6–8 weeks on average [40.8 days (1)].

A review reported 59 studies on inpatient treatment with a duration of mostly 6–12 weeks and a focus on psychotherapy in departments of Psychosomatic Medicine and Psychotherapy in Germany (3). The authors concluded that these treatments can be considered effective with a medium within-group effect size for symptom change of *g* = 0.72. However, most of the studies included in this review and meta-analysis had small sample sizes and a lack of standardized assessments.

The present study investigates the effectiveness of inpatient and day hospital treatment in departments of Psychosomatic Medicine and Psychotherapy at German university hospitals in a large representative sample employing standardized assessments. It aimed at a sample size of *n* > 2,000 to allow for sub-group analyses in patients with symptoms of depression, anxiety, eating disorders, somatoform disorders, posttraumatic stress disorder (PTSD), as well as personality dysfunctioning. A naturalistic prospective study design was chosen, (a) to obtain ecological validity, i.e., to evaluate the reality of mental health care in the field, (b) to ensure a large sample size in a multicenter study including the vast majority (76%) of German university departments of Psychosomatic Medicine and Psychotherapy, (c) to avoid the ethical problem of withholding inpatient treatment for patients in need of this kind of treatment, and (d) to avoid a selection of specific subgroups of patients for a homogenous sample as required for a randomized-controlled study.

## 2. Materials and methods

### 2.1. Study design

This study was approved by the ethics committee of the medical faculty of the Ruhr-University Bochum on October 17, 2018 (ID: 18–6388, the approval was confirmed by the ethics committees of all participating universities) and was registered in the German Clinical Trials Register (DRKS, www.drks.de; ID: DRKS00016412). Patients were recruited at inpatient and day hospital units of 19 out of 25 German university departments of Psychosomatic Medicine and Psychotherapy. Patients provided written informed consent before study entry, they were assessed by trained clinicians (physicians, psychologists), and completed a number of questionnaires. Except for baseline diagnoses all assessments were conducted three times: on admission, during the week before discharge, and at follow-up 1 year after discharge. The follow-up analyses have not been completed, yet.

### 2.2. Participants

Patients were included consecutively from January 2019 to December 2020. The departments recruited for 1 year or until at least 100 patients were included into the study. Due to the COVID-19 pandemics, recruitment was paused, when regular treatment conditions could not be maintained. Inclusion criteria were: age ≥ 18 years, sufficient knowledge of the German language, non-emergency admission for psychosomatic inpatient or day hospital treatment. Exclusion criteria were: clinically relevant organic brain disorder, and current substance dependency (excl. tobacco and prescribed medications such as benzodiazepines or opioids), and acute psychotic disorder.

### 2.3. Treatment

Several consensus driven papers describe the characteristics and standards of inpatient and day hospital treatments at German departments of Psychosomatic Medicine and Psychotherapy (2, 4, 5). Within the German health care system these standards have shaped the official definitions and cost calculations of psychiatric und psychosomatic treatment (6); e.g., defined therapeutic components are required at a minimum dosage per week. At the university hospitals best practice models usually encompass 6–8 weeks of elective inpatient or day hospital treatments. The multidisciplinary, integrative, and bio-psycho-social treatment includes psychodynamic, behaviorally oriented and systemic elements as well as components of trauma therapy. It comprises at least three sessions per week of individual and group psychotherapy; in addition, creative, body-oriented, and mindfulness-based therapies, psychoeducation, social work assistance, as well as medical and psychopharmacological treatment. The multimodal and complex treatment follows current German and international guidelines. In total, the before-mentioned components are delivered at a high dose of 15–20 h per week of individual and group treatments/ interventions. Expertise is provided by an interdisciplinary staff of health care professionals, who continuously communicate experiences, information, and reflections on every individual treatment process, which is as indispensable as regular supervision of the therapeutic team. The so called “therapeutic milieu” (7, 8) represents a crucial factor of multimodal inpatient and day hospital psychosomatic treatment focusing on psychotherapy: The (self-) reflection of the patient is stimulated in a therapeutic way by every relationship, be it to a staff member or the group of fellow patients. As a consequence, the treatment process is continued beyond the active therapies.

### 2.4. Instruments

Structured interviews were conducted to provide valid diagnosis at baseline. The Diagnostic Interview for Mental Disorders (“Diagnostisches Interview bei psychischen Störungen - Mini-DIPS”) is a German language interview for the assessment of all mental disorders comparable to the Structured Interview for DSM-IV (SCID-I) (9). The Mini-DIPS follows DSM-5 criteria and allows for conversion of the assessment into ICD-10 diagnoses to meet the requirements of the German healthcare system. The interview has been validated extensively and shows good reliability and validity (10, 11). The Structured Interview for DSM-IV axis II (SCID-II) (12) was employed in its German version for the assessment of personality disorders. In total, 22 raters were assessed for interrater reliability for SCID-II diagnoses and showed a Fleiss κ of 0.847.

A number of questionnaires were completed at baseline (T0), before discharge (T1), and after a follow-up period of 1 year (T2), the latter data point is not available, yet.

Primary outcome criteria: The German version of the Patient Health Questionnaire (PHQ-D) (13) contains three subscales to assess depression (PHQ-9), anxiety (GAD-7), and somatization (PHQ-15).

Secondary outcome criteria: Personality functioning was measured by the 12-item short version of the Structure Questionnaire of the Operationalized Psychodynamic Diagnosis (OPD-SQS; German: OPD-SFK) (14). Symptoms of acute stress reaction or post-traumatic stress disorder were measured by means of the German version of the PTSD Checklist for DSM-5 (PCL-5) (15). Finally, eating disorder psychopathology was assessed using the Eating Disorder Examination – Questionnaire (EDE-Q; German version) (16). A modified version of the Client Socio-Demographic and Service Receipt Inventory – European Version (CSSRI-EU) (17, 18) was used to quantify service utilization; Social functioning/ disability was assessed by the German 36-item version of the World Health Organization Disability Assessment Schedule 2.0 (WHODAS 2.0). CSSRI-EU and WHODAS2.0 were completed at baseline and follow-up and, thus, are not reported here (19).

### 2.5. Statistics

Descriptive statistics were employed for demographic data and diagnoses. Since the sample was not homogeneous, but naturalistic with a rather broad spectrum of diagnoses, severity of baseline pathology was used to stratify the sample. We did not focus on subgroups of categorical diagnoses, but on seven domains of psychopathology/ dysfunction. The categories were derived from the questionnaires mentioned above: Depression, anxiety, somatization, symptoms of eating disorders and trauma-related disorders, as well as personality functioning. Building on this dimensional approach, four to five subgroups within each of the seven domains were defined (from “none” to “severe” pathology); some of the questionnaires already provide a four- or five-point severity rating. Two-tailed t-tests for dependent samples were calculated for each subgroup within every domain to determine the changes during the period from admission (T0) to discharge (T1). Effect sizes for repeated measures (d_RepeatedMeasures_) were calculated according to Morris and DeShon (20).

Outcomes were compared between the subgroups of female and male patients by means of analyses of covariance (ANCOVA).

Intent-to-treat analyses were performed including all 2,094 patients. Multiple imputation was used to replace missing values of the outcome criteria. The Markov Chain Monte Carlo (MCMC) method was employed with predictive mean matching (PMM) calculating 20 imputations.

## 3. Results

### 3.1. Patients

**Figure 1:**
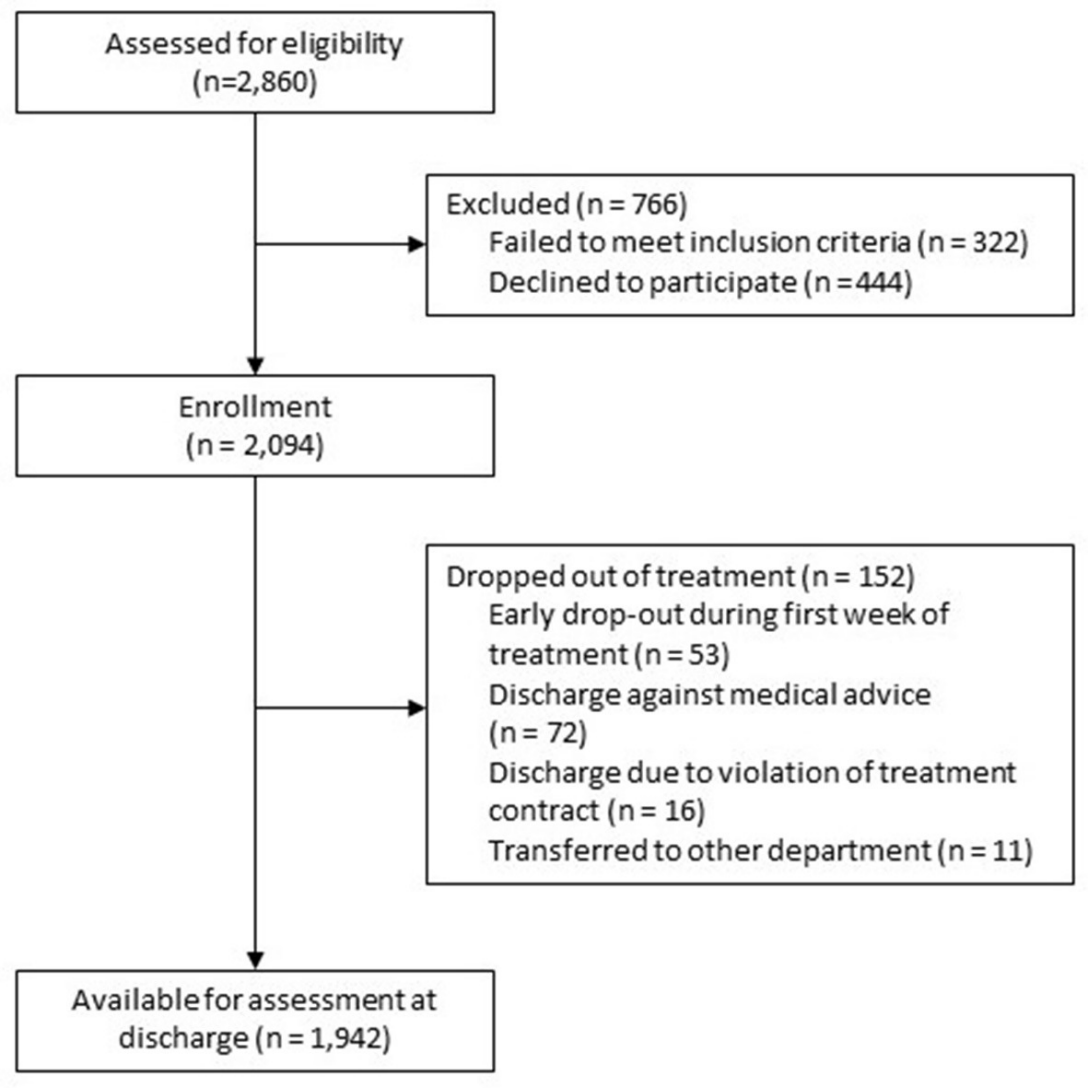
Patient flow chart.

A detailed description of the patient sample (*n* = 2,094) at baseline has already been published [21]. The patient flow chart is provided in Figure 1. In total, 610 (29.1%) patients received day hospital treatment whereas 1,342 (64.1%) patients were treated as inpatients (6.8% missing data). The average duration of day hospital treatment (including treatment-free weekends) was 46.5 (±20.2) days (range: 1–147), and the mean length of inpatient treatment was 53.8 (±23.0) days (range: 2–238). The mean age of participants at admission was 39.9 (±14.2) years, 68% of the patients were female. 48.3% lived in a partnership or were married, 47.0% had an academic degree or at least A-level. 19.6% were unemployed, 10.4% retired, the remaining patients were either working or in training. Finally, 89.6% had German citizenship.

In terms of ICD-10 diagnoses (multiple diagnoses allowed) depression (ICD-10: F32, F33, F34.1, F43.2) was diagnosed in 1,729 patients (82.6% of the whole sample); 1,079 (51.5%) patients were diagnosed with one or more anxiety disorder (F40, F41), 896 (42.8%) with one or more somatoform disorder (F44, F45), 430 (20.5%) with an eating disorder (F50), 478 (22.8%) with PTSD or an acute stress reaction (F43.0, F43.1, F43.8, F43.9), and 858 (41.0%) patients had one or more personality disorder. The majority of participants received more than one Axis I diagnosis (85%), 63.2% had three or more, and 25.8% five or more diagnoses. At least one diagnosis of a concurrent somatic disorder occurred in 1,363 (65.1%) of the patients. More detailed information about diagnoses is given in Doering et al. (21).

### 3.2. Treatment outcomes

At discharge (T1) 16% of primary outcome data were missing at random (MAR) and replaced by multiple imputation. Patients that had shown a moderate or severe psychopathology at baseline yielded clinically significant improvement at discharge in those outcome domains (Table 1). In cases of moderate or severe pathology at baseline, the effects were large (*d* > 0.8) throughout all domains of outcome. For patients with depression and anxiety the effects increased with increasing baseline severity up to *d* > 2 in anxiety and *d* > 3 in depression. Patients with the highest severity in eating disorder pathology and symptoms of PTSD revealed less symptom reduction than those with moderate severity. Compared to the other domains, changes in somatization and personality functioning showed the lowest effect sizes, nevertheless up to *d* > 1.1 in the groups with severe baseline impairment. In almost all domain patients with no/subthreshold baseline symptomatology showed a slight increase of symptom severity.

**Table 1:** Outcome measures – changes between admission (T0) and discharge (T1) (*n* = 2,094) [two-tailed paired t-tests, effect sizes (d) for repeated measures (20)].

	***N (%)***	**Pre (T0 = admission) *mean (SD)***	**Post (T1 = discharge) *mean (SD)***	**T**	**df**	***p***	***d***
**Depression (PHQ-D), sum score**							
None (< 5)	99 (4.7)	2.78 (1.37)	3.92 (3.49)	−3.397	98	< 0.001	−0.70
Mild (5–9)	331 (15.8)	7.29 (1.33)	6.15 (3.51)	5.820	330	< 0.001	0.66
Moderate (10–14)	529 (25.3)	12.05 (1.38)	8.33 (4.16)	20.460	528	< 0.001	2.07
Moderately severe (15–19)	627 (29.9)	16.99 (1.39)	11.23 (5.11)	27.991	626	< 0.001	3.10
Severe (≥20)	499 (23.8)	22.32 (1.94)	14.93 (5.79)	30.276	498	< 0.001	3.31
**Anxiety (GAD-7), sum score**							
None (< 5)	181 (8.6)	2.58 (1.29)	3.35 (2.66)	−3.884	180	< 0.001	−0.49
Mild (5–9)	554 (26.5)	7.21 (1.38)	5.72 (3.50)	10.024	553	< 0.001	0.86
Moderate (10–14)	694 (33.1)	12.01 (1.44)	8.11 (4.26)	23.978	693	< 0.001	2.08
Severe (15–21)	656 (31.3)	17.41 (1.90)	10.87 (5.22)	32.797	655	< 0.001	2.79
**Somatization (PHQ-15), sum score**							
None (< 5)	115 (5.5)	3.21 (1.11)	4.05 (3.09)	−2.941	114	0.004	−0.60
Mild (5–9)	476 (22.7)	7.37 (1.39)	6.96 (3.32)	2.797	475	0.005	0.25
Moderate (10–14)	664 (31.7)	12.02 (1.36)	9.98 (4.04)	13.545	663	< 0.001	1.25
Severe (15–30)	830 (39.6)	18.70 (3.04)	15.11 (4.92)	23.450	829	< 0.001	1.15
**Eating disorder pathology (EDE-Q), global mean score**							
None (< 1)	824 (39.4)	0.32 (0.29)	0.35 (0.47)	−1.633	823	0.103	−0.09
Mild (1–1.99)	415 (19.8)	1.42 (0.30)	1.24 (0.91)	4.154	414	< 0.001	0.50
Moderate (2–2.99)	307 (14.7)	2.44 (0.28)	2.16 (1.01)	4.776	299	< 0.001	0.78
Moderately severe (3–3.99)	283 (13.5)	3.43 (0.28)	2.66 (1.06)	12.880	282	< 0.001	2.37
Severe (≥4)	263 (12.6)	4.82 (0.57)	3.81 (1.14)	14.280	262	< 0.001	1.44
**Symptoms of PTSD (PCL-5), sum score**							
None (< 30)	961 (45.9)	15.01 (9.24)	14.42 (12.89)	1.467	960	0.143	0.06
Mild (30–39)	351 (16.8)	34.55 (2.82)	25.62 (14.79)	11.611	350	< 0.001	2.55
Moderate (40–49)	295 (14.1)	44.40 (2.84)	34.25 (15.78)	11.166	294	< 0.001	2.74
Moderately severe (50–59)	264 (12.6)	54.01 (2.94)	39.63 (17.25)	13.541	263	< 0.001	3.61
Severe (≥60)	214 (10.2)	66.82 (5.52)	52.22 (16.07)	12.779	213	< 0.001	1.92
**Personality functioning (OPD-SFK), sum score**							
None (≤ 10)	169 (8.1)	6.49 (2.95)	7.62 (6.02)	−2.719	168	0.007	−0.36
Mild (11–20)	498 (23.8)	16.15 (2.76)	14.85 (7.06)	4.202	497	< 0.001	0.39
Moderate (21–30)	781 (37.3)	25.70 (2.82)	22.59 (7.86)	11.245	780	< 0.001	0.88
Severe (≥31)	637 (30.4)	36.64 (4.39)	30.91 (8.41)	18.555	636	< 0.001	1.19

### 3.3. Gender differences

All outcome analyses were calculated separately in women and men. A few group differences occurred – all of them in favor of men, who had larger improvements, however, not to clinically relevant degree. Significant group differences (ANCOVA) occurred in the strata of moderate moderate somatization, no symptoms of PTSD, and no impairment of personality functioning (*p* < 0.05). Tendencies toward differences were found in mild and severe somatization, moderate PTSD symptoms, as well as moderate and sever impairment of personality functioning (*p* < 0.01).

## 4. Discussion

This is by far the largest prospective and naturalistic inpatient/day hospital study in the field and the first multicenter study that includes the vast majority of German university departments of Psychosomatic Medicine and Psychotherapy. Inpatient and day hospital treatment in departments of Psychosomatic Medicine and Psychotherapy is effective and produces high within-group effect sizes for the improvement in different outcome domains, i.e., depression, anxiety, somatization, eating pathology and PTSD, as well as personality functioning.

Liebherz and Rabung (3) included 59 studies on inpatient treatment in departments of Psychosomatic Medicine and Psychotherapy in their comprehensive review and reported a medium overall effect size for symptom change at discharge of *g* = 0.72; for change in interpersonal problems they found a small effect size of *g* = 0.35. These comparably low numbers can be attributed to the fact that almost all of the studies included were naturalistic cohort studies with mixed diagnoses. As a consequence, psychopathology at baseline varied substantially and floor effects occurred due to those patients, who had no impairment in different outcome domains at baseline. A patient with, e.g., an eating disorder might not have had any depressive symptoms on admission and, thus, no change in depression during the treatment. When all diagnostic groups are included in the same analysis, the changes of specific outcome criteria will probably be erroneously low. This could be the reason for the fact that within-group effects in randomized-controlled trials (RCT) are higher since they usually feature diagnostically homogeneous samples with moderate to severe symptom levels.

To avoid these floor effects we used a different strategy and calculated the effects for 4–5 groups of severity at baseline for every outcome domain – some instruments, like the PHQ-D already provide this categorization. As a consequence, the effect sizes are considerably higher than in other studies with mixed samples. Our results should be compared to the within-group effect sizes of RCTs with homogeneous samples with a baseline severity corresponding to the respective subsample of this study.

Another issue has to be kept in mind when dealing with these effect sizes: There is an ongoing debate whether observational studies should deduct a certain effect for spontaneous remission that would also have occurred in a sample without any treatment. Grawe et al. (22) reported an average effect size of *d* = 0.1 for untreated control groups in psychotherapy outcome studies, an estimate that was confirmed by Leichsenring and Rabung (23), who reported *d* = 0.12. We decided to report the effect sizes we actually found in our samples. However, to determine the improvement due to the treatment in comparison to no treatment, the effect sizes should be corrected by *d* = 0.1 to 0.15.

The effect sizes of the improvement of depressive symptoms varied between *d* = 0.66 in patients with mild symptoms and *d* = 3.31 in those with moderately severe symptoms. In their mixed samples from studies in departments of Psychosomatic Medicine and Psychotherapy, previous studies reported effect sizes of 0.43 to 0.93 (24–28) and 1.66 in one case (29). The treatment regimens of these studies are comparable to those in our study. However, the samples were mixed and the analyses included all patients regardless of their baseline levels of depression. A recent meta-analysis on inpatient treatment for depression in departments of psychiatry and psychotherapy revealed a mean between-group effect size of *g* = 0.24 (30). Within-group effect sizes of these studies were, e.g., *d* = 1.91 for Interpersonal Therapy (IPT) (31) and *d* = 2.1 for cognitive behavioral therapy (CBT) (32). The patients of these studies showed a level of depression comparable to our “moderately severe” and “severe” groups, however, the treatment programs were highly specialized and shorter (5–6 weeks); the longer duration as well as the multidisciplinary and multimodal approach will probably explain the considerably higher effect sizes in our study.

The treatment effects on symptoms of anxiety were similar to those of depression. In the most severe patient subgroups the effect sizes were above *d* = 2. When compared to the above-mentioned studies from individual departments delivering inpatient treatment with effect sizes between *d* = 0.4 and 1.0 (24–28), the effects of this study again were much higher probably for the same reasons discussed above. A large observational study on inpatient psychosomatic treatment reported effect sizes of *d* = 0.71 to 0.88 for the decrease of anxiety scores in patients with anxiety disorders (33). A recent meta-analysis of outpatient psychotherapy (34) revealed effect sizes to be around *d* = 1.4. Since higher effects were found for the most severe patient groups in our study, it can be assumed that the lower effects of the outpatient studies can be attributed to a lower severity of illness at baseline in addition to the lower treatment intensity.

The improvement of somatization symptoms was lower than that of depression and anxiety. For the two more severely affected subgroups of patients we found effect sizes of *d* = 1.25 and 1.15. These numbers are close to those of previous studies on the inpatient treatment for somatization, which varied between *d* = 0.3 and 1.0 (24–29, 35). A recent meta-analysis on short-term psychodynamic (outpatient) psychotherapy reported a mean effect size for the reduction of somatic symptoms of *d* = 0.84 (36), another meta-analysis on psychological interventions for patients with medically unexplained symptoms reported a mean effect size of *d* = 0.6 (37). The fact that higher symptom severity predicts somewhat smaller positive effects has been reported in a recent meta-analysis (38). A general overview of the efficacy of different treatment approaches is given by Henningsen et al. (39). As a limiting factor for the interpretation of this result, it has to be taken into account that in our sample almost two thirds of the patients suffered from comorbid somatic disorders, which might have affected the self-ratings of somatization.

A similar pattern was found for the improvement of eating disorder pathology. While mild and moderate severity subgroups achieved medium high effects between *d* = 0.5 and *d* = 0.78, the effect size of the most severely disordered patients was somewhat lower (*d* = 1.44) than that of moderately severe patients (*d* = 2.37). It has repeatedly been reported that particularly anorexia nervosa, but also bulimia nervosa are difficult to treat with remission rates of approximately 50% for both eating disorders (40–44). This might be particularly true for the most severely disordered patients. We assume that they would need longer treatments than the average of 8 weeks delivered in this study. A meta-analysis reported a mean (within-group) effect size of weight gain in anorexia nervosa during treatment of *d* = 1.22 for intent-to-treat analyses and 1.68 for per protocol analyses (45). For bulimia nervosa, a recent meta-analysis of outpatient psychotherapy yielded an average (within-group) effect size for self-reported eating pathology of *g* = 1.35 (46).

The changes in symptoms of PTSD showed a similar pattern to that in eating disorder pathology. The most severely affected subgroup revealed lower effects than mildly and moderately affected subgroups (*d* = 1.92 vs. *d* = 2.55 to 3.61). A meta-analysis (47) showed a mean effect size for all treatments of *d* = 2.14. Recently, an effect size of *d* = 1.30 for an inpatient treatment in a German university department of Psychosomatic Medicine and Psychotherapy was found (48). As mentioned before, we would assume that the most severely affected patients, who frequently show complex traumatization and comorbid personality disorder, might need longer and more specific treatments than those offered across different departments of Psychosomatic Medicine and Psychotherapy.

The changes in personality functioning similar to somatization were clearly lower than those of the other domains. This could be expected, since personality functioning is bound to personality traits to a much higher degree than the psychopathological symptom domains reported above. Nevertheless, the effect sizes for the moderately and severely dysfunctional subgroups were still large (*d* = 0.88 and 1.19). So far, few studies investigated changes in personality functioning during treatment. For inpatient treatment, effect sizes of *d* = 0.31 (49), *d* = 0.52 (50), and *d* = 0.68 (51) were reported. Doering et al. (52) found an effect size of *d* = 1.0 in their outpatient psychotherapy study for borderline personality disorder; however, it has to be taken into account that these treatment studies employed disorder specific treatment models in a homogenous sample different from the mixed sample in the MEPP study reported here.

In all outcome domains those patients, who did not show any relevant pathology in that domain at admission, yielded an increase of symptoms during treatment. These sub-threshold changes can be interpreted as a regression to the mean. However, in some patients an increased awareness of mental (emotional) and physical states might have occurred because of the treatment, which could be regarded as an improvement rather than a treatment failure.

The comparison of outcomes in women and men revealed a few significant differences. Remarkably, all of them showed a higher degree of improvement in men. Although, none of these differences appeared to be clinically significant regarding group means, individual female patients might have had considerably less favorable outcomes. As a consequence, it is of utmost importance to investigate whether the characteristics of pathology in females differs from that in men, of whether treatment concepts might be more suitable for male patients.

The major strength of this study is the large sample size of *n* = 2,094, the prospective study design, and the structured diagnostic assessment by means of interviews. A limitation is the lack of a control group. The problem of diagnostic heterogeneity of the sample was dealt with by creating subgroups of different symptom severity at baseline for each outcome criterion – the sample size allowed for sufficiently large subgroups to perform separate analyses. When compared to the sample sizes of other treatment studies, this has to be taken into account. In addition, a certain effect of spontaneous remission (*d* = 0.1 to 0.15) should be deducted from the reported effect sizes.

Taken together, this large prospective observational study indicated significant positive outcomes with high effect sizes, particularly in those domains where moderate and severe psychopathology was present at baseline. Thus, it can be assumed that treatment with a focus on psychotherapy in German university hospitals of Psychosomatic Medicine and Psychotherapy is highly effective.

## Data availability statement

The datasets presented in this article are not readily available because the European General Data Protection Regulation (GDPR) does not allow to share personal data of patients publicly (https://gdpr.eu). The ethics commissions of all of the study centers have approved the study under the condition that even the transfer of data from the German sites to the Austrian PI (SD) can only take place according to specific security regulations. Requests to access the datasets should be directed to stephan.doering@meduniwien.ac.

## Ethics statement

The studies involving human participants were reviewed and approved by Ethics Committee of the Medical Faculty of the Ruhr-University Bochum on October 17, 2018 (ID: 18–6388). The patients/participants provided their written informed consent to participate in this study.

## Author contributions

After an initial conceptualization of the study design by SD and SH, all authors contributed to the planning of the details of the study design and logistics as well as to the data collection. SD, SH, and HK performed the data analysis and wrote the first draft of paper. All authors contributed to the article and approved the submitted version.
